# Abstracts and Gleanings

**Published:** 1882-05-20

**Authors:** 


### ABSTRACTS AND GLEANINGS.

**The Treatment of Syphilis without Mercury—A New Abortive Method.**—Dr. J. Edmund Guntz, of Dresden, in a work just published by him, makes some novel announcements regarding the treatment of syphilis. If true, they are of the highest importance, for he claims to be able "not only to do away with mercury in syphilis, but in a large proportion of cases to abort the disease."

It is now over twelve years since Dr. Guntz first wrote on this subject. He is, therefore, not a novice in the matter. In 1869 he advocated the use of bichromate of potassium as being a useful drug in treating syphilis.

He could not prove any very great advantages for it, however, at the time. It acted slowly and was apt to disturb the stomach, but being convinced that there was something in the drug, he set to work to find some way of getting more into the system without producing functional disturbance. For a time he combined the bichromate with the nitrate of potassium, and gave pills containing about 1-15 gr. of each three times a day. With these pills he produced "remarkably favorable results." Yet the action was slow, and a prompt amelioration of symptoms was needed, as in malignant cases the remedy would hardly meet the expectations.

From the favorable results obtained by giving the various minerals in solutions with carbonic acid water, our author was led to attempt administering chromium in the same way, and with, as he now claims, very great success. He found that much larger doses could be taken in this form, and that a profounder impression on the system could thus be made. As a maximum dose he was able to give three and a half grains (.3 grammes) daily of bichromate of potassium in about 600 grammes of carbonic acid water, this being divided into five doses. Larger amounts provoked vomiting.

This "chromwater," as he calls it, could also be given daily for weeks and months in all forms of syphilis without detriment to the health.

Having described his method of giving the drug, Dr. Guntz discusses its action upon the initial stage of syphilis and upon the disease itself after its full development in the system.

In estimating the possible value of any drug as an abortive of syphilis, the numerous sources of error are referred to. The existence of and difference between true chancre and chanchroid are admitted.

The following are his statistics:

Within one and a quarter years the author treated 194 cases of chancre. For a comparative study he selects only 85 of these, since in the others there were sources of error. In 14 of these 85 cases the sores were cauterized. The remainder were treated with nothing but the chromwater; and in 47 of them constitutional syphilis failed to appear. In order to avoid every possible chance of mistake, the author excludes 10 of this 47. Even then there were left 37 patients, or over one-half, who, when given chromwater alone, developed no after-symptoms. It is not stated, however, how long they were watched, except that 18 were under observation for 159 days.

Still more favorable results took place with the 14 cases in which the initial lesion was cauterized. Of these only two developed symptoms of constitutional syphilis.

Of the 85 patients, therefore, presenting, as Dr. Guntz asserts, initial leisons of syphilis, 49, under the "chromwater" treatment, remained entirely free from the disease. This is certainly a very extraordinary showing, and will be received with a great deal of incredulity.

If this new agent is given after constitutional symptoms make their appearance, its action is to ameliorate the disease and hasten its course. It is efficient even in cases where mercury fails, and it acts more pleasantly and promptly. In fact, the disease is "in the shortest time definitely cured."

The author has, for several years, used the chrome salt exclusively in the treatment of syphilis, and has given it in more than a thousand cases. He has recorded the histories of a large number of his cases.

Dr. Guntz has also used his chromwater with the best results in diphtheria.

He suggests that the drug acts by reason of its powerful oxidizing properties. Without committing himself to any germ theory, it is thought that there is certainly a specific poison which develops in the various contagious diseases. And in chromium we have an agent that is not inimical to the system itself, but rather benefits it.

The importance of Dr. Guntz's claims, and the caution with which they should be received, are alike apparent and need no comment.—*Medical Record.*

**Fractures of the Patella.**—M. Pinsot, in a review of the subject of operative interference of these fractures, draws the following conclusions:

1. Puncture of the joint should be practiced in all cases where there is much effusion into the articular cavity; it should be immediate, and it is not necessary to follow it by drainage.

2. After the puncture, and in cases where the ordinary apparatus are insufficient to maintain coaptation of the fragments, suture of the divided patella may be practiced, as recommended by Kocher.

3. In all cases the apparatus should be examined very frequently for the first few days, until the articular swelling has subsided.

4. For several months after the union of the fracture the limb should be provided with an apparatus limiting flexion.

5. The opening of the articulation with osseous suture is suited to cases in which puncture is not sufficient to remove the articular exudation.

6. It is necessary also in pesendarthroses and in cases where an excess of callus interferes with the motion of the joint.—*Revue de Chirg.—Amer. Jour. Med. Science.*

**Treatment of Pneumonia.**—I wish to draw attention to the remarkable effects produced by the perchloride of iron, combined with hydrocyanic acid, in cases of pneumonia of a low type, especially those due to blood-poisoning. Most practitioners will agree in having seen cases of pneumonia run a course so like, in its general aspect, that of erysipelas, as to lead them to imagine that they might be due to a similar cause, taking effect in the interstitial substance of the lung, instead of in the subcutaneous tissue. I have seen many such, and I have begun to apply a similar treatment, with, as I say, truly marvelous effects. The first case of the kind, in wich I ventured on this treatment, was that of Mrs. G., aged 35, who had double pneumonia, with pleurisy on the right side, in February, of last year. When I first saw her, the pulse was 140, the temperature in the axilla 103°, and the sputa of a deep rust color. I ordered mustard and linseed poultices, and the following mixture:

℞. Liquoris ferri perchloridi fort................ ʒ ij.
Acidi hydrocyanici (Scheele).............. ♏ viij.
Aquam ad.................................. ℥ viij.
M. Two teaspoonfuls to be taken every hour, with an intervening teaspoonful of brandy in water.

After thirty hours, the pulse had fallen to 100, the temperature to 99°, the sputa were entirely devoid of blood, and the breathing was almost normal. This patient made a rapid recovery.

In the last case of the kind, coming under my notice, which occurred last week, the patient seemed to be in a state of collapse, or syncope, the pulse was 144; the breathing in short gasps; the finger-ends, as seen through the nails, of the color of a thunder-cloud; and both lungs in a general state of clog. Delirium also lasted a whole night. She had complained of shortness of breath, and had a phthisical aspect and family history, but had never had any cough until the present time. I ventured upon the same treatment with her; and her pulse is now 96, temperature all but normal, sputa devoid of blood or discoloration of any kind, and she herself anxious to get up.—*D. Biddle, Kingston-on-Thames.*

**Blood Enemata.**—Dr. Sansom thus writes of the employment of blood in nourishing enemata: Ox blood is usually employed, but sheep's blood may be used. It is necessary that it be defibrinated the moment it is drawn. Butchers understand this process, and will supply what is called "whipped" or "stirred" blood. It is, of course, requisite that the blood be fresh—that it be not kept more than a single day. In urgent cases, where there is no stomach digestion, two or three ounces of blood may be injected into the rectum every two or three hours; the fluid may be warmed by placing the containing vessel in hot water, but it is often borne equally well when cold. For chronic cases in which it supplements stomach alimentation, it is administered in quantities of from two to six ounces twice a day. In some cases it tends to promote constipation; in a very small percentage, the opposite condition of irritability.—*The Lancet.*

**Delirium Tremens.**—At a recent meeting of the Cambridge (Eng.) Medical Society (Lancet), Dr. Latham introduced the subject of the treatment of delirium tremens. He thought the subject offered scope for considerable difference of opinion, and was a suitable one for general discussion. He referred, first, to sleeplessness as a prominent symptom, and said that if sleep were obtained, recovery generally ensued quickly. Was the induction of sleep, then, a *sine qua non* in treatment? If sleep did not ensue, subarachnoid effusion and coma were likely to follow in some cases, while the majority might recover; but Dr. Latham thought the disease ought to be treated actively, and that dangerous remedies might be used with advantage to patients. He divided cases of delirium tremens into three classes: (1) Patients in moderate health who had used stimulants in excess; (2) those in robust health who had indulged after excitement or distress, chiefly young men; and, (3) those broken down in health, and with damaged organs. The first class he thought could be treated with opium, without risk, provided the urine was free from albumen; and he had been surprised to find albumen in the urine in a large proportion of cases without any other indications of kidney disease, and the albumen disappearing as the patient recovered. If albuminuria existed, the use of opium or morphia could only be pernicious. The first thing to do was to give soup, beef-tea, and alcohol in nearly the accustomed doses for twenty-four hours, and then opium; having previously given, if necessary, a dose of calomel. He advised the hypodermic injection of morphia, first in half-grain doses, and then in doses of one-quarter-grain every half hour till sleep was procured or the patient was distinctly under its influence, as shown by the condition of the pupil. He believed diminution in quantity of stimulants taken by the patient to be an early symptom, and not a cause of the disease. In the second class he advised, not opium, but henbane. He had given bromide of potassium in forty-grain doses every four hours, and found the tincture of henbane in doses of one drachm every four hours succeeded better. He also thought that Merck's preparation of hyoscyamine would be useful, but had not yet given it a trial. In the third class he thought opium should be given with great caution, and that free administration of stimulants was important, even as large doses as those customary with the patient. He mentioned a case in which a gentleman, in his eager desire for insensibility, had taken 240 grains of chloral and the same quantity of bromide of potassium at a dose, and survived after very free action of the skin, bowels, and kidneys. He deprecated the use of large doses of digitalis, as suggested by Dr. Jones, of Jersey. Mr. T. Hyde Hills, speaking from his experience as a jail surgeon, thought leaving off stimulants was a cause of the disease, and that many prisoners became delirious after admission, owing to this deprivation. He advised giving the usual amount of alcohol to which the prisoner had been accustomed, and thought chloral of benefit. Mr. Hodson thought that the combination of chloral and capsicum was useful. Dr. Smith alluded to the usefulness of cold sponging of the body, as a means of inducing sleep. Dr. Latham, in reply, stated that he recommended the capsicum in confirmed cases; it was much esteemed in India. In young men he approved of Dr. Graves' plan of giving antimony and opium every two hours.—*Med. and Surg. Reporter.*

**Ozone as a Sleep-producing Agent.**—Prof. C. Binz, in a series of articles contributed to the Berl. Klin. Woschenschrift, announces the discovery of nerve-depressing and sleep-producing properties in ozone.

The accepted view regarding this gas has been that it is very easily decomposed, nascent oxygen being set free; that it is extremely irritating on this account to the tissues, acting much like chlorine, and that it cannot be absorbed by the blood. Binz, however, shows that in proper quantities it is not irritating, can be inhaled and absorbed, producing, as he claims, peculiar effects on the nervous system.

The experiments were tried upon human beings. Dr. Hugo Schultz was the first to submit himself. Subsequently five other gentlemen inhaled the gas. Three of them were put to sleep by it, the others were slightly stupefied or otherwise depressed. The time required for bringing on sleep varied between six and sixteen minutes. The sensations during this time were very agreeable. After removal of the gas the sleeper would awake within half a minute, generally sooner. It was suggested that in one quite susceptible person the condition was a hypnotic one, but inhalation in the same way of pure air produced no effect. After awaking, there was some feeling of fatigue, but this soon passed away.

Large and prolonged doses of the gas produced sensation of nausea, dizziness, and strangling; but the diluted ozone was breathed for over half an hour without harm. Binz states that in too small amounts no effect is gotten; in too large ones irritation is produced. He compares its action in this respect to that of alcohol when given. Prof. Binz claims no practical results from his discovery as it stands at present, but thinks that like every new scientific truth it may have eventually some useful bearing.—*New York Med. Record.*

**Deaths from Chloroform.**—Within a few weeks two patients have died in Chicago from the effects of chloroform administered for the purpose of having teeth extracted. The last case occurred December 20th. The patient was in a dentist's chair, and a physician administered the anæsthetic. Thirty minutes were required to anæsthetize the patient, and from an ounce and a half to two ounces of chloroform were administered. The patient became cyanosed and ceased to breathe after two or three teeth had been extracted. He was a man about forty years old, and was supposed to be healthy, although neither the dentist nor physician had ever seen him before or even knew his name or residence. The patient was in a sitting posture during the anæsthesia and operation.—[The Boston Med. and Surg. Journal.

**Do Southern Hogs Have Trichinæ?**—An investigation, which seems to show that southern hogs do not have trachinæ, was made by Dr. Jansen T. Payne last summer. His report was submitted to the American Public Health Association at its last meeting. In six months Dr. Payne examined 5,400 hogs, finding only 22 infected with the parasite in question. The infected animals were reported as having been received from the following places: St. Louis, 18; Louisville, 2; and from the West, marked "unknown" 2; making the total of infected hogs, 22. Of the hogs examined, only 529 came from St. Louis; most of them came from Louisiana (2,473) and Tennessee (1,060).

The observations lead to the belief, therefore, that southern-bred hogs are free from trichinæ. Still, such a deduction is not absolutely safe. If the fact were really proved, it would be one of great advantage to southern pork-raisers. Even as it is, they can profit by the fact that Tennessee and Louisiana hogs are almost entirely free from disease.

Incidentally, some other facts regarding the origin and communicability of trichinosis were developed. Observations seemed to show that hogs infect each other when enclosed in the same pen, and do not depend upon the rat as an intermediate host. The parasite is passed out of an infected animal along with undigested food, is then eaten by a sound hog, who in turn becomes infected.

By Dr. Payne's examinations it was also ascertained that all the hogs infected with trichinæ were corn fed animals. No mast-fed animals was found to be infected.—*Journal of Comparative Medicine.*

**Sulphur for Pimples on the Face.**—Dr. Gage Parsons believes that Mr. Erasmus Wilson was the first to propose sulphur lotion in acne punctata, according to the Practitioner. The usual lotion of the flowers of sulphur with glycerine and water is undoubtedly a valuable remedy, but from the readiness which the sulphur separates it is inelegant and inconvenient, while it is not quite satisfactory in its results. A far more efficacious mode of using sulphur is to dust the face with pure precipitated sulphur every night with an ordinary puff used for toilet purposes. Recently two severe cases of acne of two year's standing, which had resisted the ordinary method of treatment, yielded at once to sulphur thus applied. If the sulphur be scented with oil of lemon or roses, it will form an elegant cosmetic.—[Can. Med. Rec.

**Earache.**—"In the course of practice you will often be called upon to attend a case of earache. This means, pathologically speaking, acute inflammation of the membrana tympani. Now, in such cases you may quickly subdue the inflammation, relieve the patient from the excruciating pain he is suffering, and save him, perhaps, from subsequent confirmed deafness. The treatment, from which such a desirable result may be obtained, is similar to that which you will find so beneficial in analogous cases of eye diseases—viz: leeches behind the ear, hydrag. c. creta and belladonna powders, with warm fomentations."—*Wharton Jones, in London Lancet.*

**To Remove Foreign Bodies on the Conjunctiva Beneath Upper Lid.**—Dr. Roosa, in New York Medical Record, says:

In the first place, the patient, so far as possible, should be in a comfortable position. He may be seated in a chair with the head well supported. With a foreign body in the conjunctival sac the patient is in distress, and his eyeball is not entirely under his control. He is not as able to respond to your requests as to where he should look as though it were not there. Get the head, therefore, properly supported, and then with the thumb press the integument of the lid against the eyebrow, so as to put it upon the stretch, and tell the patient to look down—not to turn the head down, but to turn the eyes down. Then catch the eyelashes and edge of the lid with the fingers of the other hand and turn the lid quickly over the thumb; in the meantime the patient looks down constantly. You see how readily, by this simple manipulation, I have succeeded in exposing our friend's conjunctiva, which we find to be hyperæmic in a marked degree, and yet, unless you have practiced these movements somewhat, you will be mortified when you make your first attempt to perform this simple operation with lookers-on around you. An intelligent assistant will be of advantage, and there is scarcely any place in which you cannot secure one to hold the head, and his determination must be that the patient shall not turn his head down, but shall look down, and thus turn the eyes downward.

Next, how are you to remove the foreign body after you have found it? How shall you get rid of that little black speck which has been the cause of so much discomfort to the patient—which, perhaps, has given him a very uncomfortable night, has concentrated all his attention, and almost led him to curse railroad carriages? It is usually a simple matter after having everted the lid, but there is method even here. If it is a lady who has thus been selected, she will have her own handkerchief in her pocket, and she will feel better satisfied if you will use that rather than your towel, for she will not feel sure that the towel is clean, while she knows that the handkerchief is.

Remove the foreign body with the handkerchief, then, and do not throw it away. Show it to the patient, and be sure that he or she recognizes it—and why? The little shallow depression which that foreign body made in the conjunctiva will give a sensation of discomfort, and sometimes, not always, give rise to the same symptoms that existed before the foreign body was removed. It is well, under such circumstances, to have the proof of what you have done, lest the patient say, "He said he took it out. I paid him for removing it, but I know it is in there yet."

In the majority of cases, however, the feeling of comfort is so great, the relief is so instantaneous, that there is no occasion for such unpleasant complaint.

You will seldom need any instrument for removing a foreign body from the conjunctival sac, beyond the hands and a delicate piece of cambric or a smooth, well-worn towel. The patients very seldom need any after treatment. It is well to direct that the eye be bathed with warm or cold water, and protected from dust, and the blood-vessels will soon return to their normal condition. I should mention that occasionally a patient will return, insisting that a foreign body is in the eye, when there is none there. A few evenings since, a medical gentleman came to me, saying, "I have something in my eye, and I am suffering very much. My friend, Dr. ——, says there is nothing in it, but I know that there is." But I was unable to find it, and the man's indignation was very great, and he exclaimed, "I know it is there, for I feel it." I then examined his eye very carefully again, after which I said to him, "You may be certain that there is no foreign body in your eye." I first examined his eye by the aid of ordinary light, but when he was so positive with reference to the presence of the foreign body, I took him to a gas-light and with a two inch lens, threw the concentrated rays into his eye, and examined the entire conjunctival space, and then I was unable to answer him in the most assuring manner. What was the cause of his sensations? An attack of conjunctival catarrh was beginning. Many of those cases begin very suddenly, as though something struck or entered the eye, and you must not be deceived and take it for granted that there is a foreign body in the conjunctuval sac simply because a patient has a sensation as though some rough substance is pressing upon the cornea.

**Ergot and Opium in Obstetrics.**—Put about two drachms of spurred rye in a pipkin, on which pour half a pint of boiling water, cover and place it before the fire, and let it simmer until it assumes the color of strong tea; then strain, and mix one and a half drachm of tinct. of opium. B. F. Give a patient about half of the mixture, and if necessary, give the remainder in half an hour afterwards; although it very rarely happens that a second dose is needed. It is better not to administer the above until the os is well dilated Since using the above mixture in midwifery, Dr. Samuels says he has not had one fatal case of the child, nor a case of hour-glass contraction of the womb, so frequently met with, while giving ergot alone—nor an unmanageable case of asphyxia in the child. The above combination has also proved successful with the use of the forceps, even in a threatened case of post-partum hemorrhage. Dr. Samuels says he has used opium with ergot in about five hundred cases, and in each good results followed, whether in labor cases or flooding, or from other causes.

He also speaks of having noticed that the late Sir James Y. Simpson, of Edinburgh, on one occasion administered a vapor of ether and ergot, for the same purpose as he prescribed his formula, with beneficial results.—*Bradford, E. C. Med. Journal.*

**A Prize of $1000 in Waiting.**—Some few years ago a prize of $1000 was offered by a Boston physician for the best essay on "The probability of the Discovery of the Cure for Malignant Diseases, and the line of Study and Experimentation likely to bring such a Cure to light" Three essays were presented, neither of which was deemed worthy by the judges. Dr. J. C. Warren, representing the donor, announces that the question is still open and the money remains on deposit for the successful competitor. Essays must be presented not later than December 1st, 1883. The decision will be made chiefly from a practical stand-point, the object of the donor being to obtain suggestions by which a search for the cure for cancer may be instituted. The amount of the prize is commensurate with the magnitude of the subject, and ought to bring forth fruit if there is a reasonable share of literary ambition in the profession. We believe it is the largest sum ever offered in America as a prize for a literary or scientific production.—*Pacific Med. and Sur. Journal.*

**Treatment of Infantile Diarrhœa.**—This is the chief disease of infants, and the hygiene is more necessary very often than the medicines employed. Among infants at the breast, the cause of diarrhœa is as often the fault of the nursing mother as of the infant. The abuse of baths is a prime cause, being often more injurious than beneficial, from being too prolonged, instead of a single washing of the body.

As to dentition Dr. Simon attributes great influence, though denied by many authors. In the simple cases, after ascertaining the cause, if possible, stop everything but the milk and give a spoonful of coffee with a little alkaline water. A tepid bath should be given each day and a starch injection; every day at each meal give a portion of the following powder:

| | | |
|---|---|---|
| ℞ Calcined magnesia, | | 10 grammes, |
| Prepared cream | } | aa 2 grammes. |
| Sub. nitrate of bismuth | } | |

At last apply warm fomentations to the bowels.

If the diarrhœa coutinues and becomes catarrhal, *i. e.*, accompanied with a considerable secretion and intermitting fever, it is better to give a vomit and afterwards a portion of subnitrate of bismuth, 4 grammes with one drop of laudanum, to an infant under one year of age.

At the advanced stage the diarrhœa becomes sympathetic of enteritis. This is distinguished by the passages, which are green, acid and extremely irritating. The fever is persistent and the countenance indicates suffering, a sign very significant of unfavorable results.

It is now necessary to give laudanum, the true treatment of enteritis, with sub-nitrate of bismuth, a drop of the former to 60 grains of the latter, to each year of age. Paregoric can be used in the place of the laudanum—5 drops for 1 of laudanum. It ought not to be forgotten to continue the laudanum after the passages are checked, but in diminished doses. If vomiting should occur, prepared chalk and lime-water should be applied, and a small blister over the epigastric region, using the necessary precautions.

In enteritis it is often observed that membranous fragments are expelled, with violent colic, and in these cases the injections should be frequent and cathartics used. It is necessary to keep up this treatment with alkaline waters, excluding grease and indigestible articles, and hydrotherapy, when the age of the infant permits.

Chronic enteritis is extremely difficult to treat; opiates and astringents should be used and afterwards revulsives, as tr. iodine, croton oil, and vesicatories.

The treatment of diarrhœa is of great importance, because it may be but the beginning of choleraic diarrhœa. In these cases the danger is imminent. In these cases give a spoonful of coffee and Malaga wine, and coffee and brandy. If possible, a wine bath should be given; this stimulates the functions of the skin, and should only last five minutes.—*Jules Simon, in Journal de Therapeutique.*

**Sudden Death During Forced Depression of Tongue.**—A woman, sixty years old, suffering from tinnitus aurium and partial deafness, applied to Dr. Moure, who ascertained the existence of catarrh and obstruction of both eustachian tubes. Wishing to examine the pharynx, he directed the patient to open her mouth; the tongue being in the way, he introduced a depressor. No sooner had he depressed the tongue than the patient drew a hissing inspiration, and commenced suffocating. Believing he had to deal with a spasm of the glottis, the Doctor had recourse to artificial respiration, but asphyxia increased, rales set in. Tracheotomy was then practiced, but asphyxia continued, spumous blood was discharged through the cannula and by the mouth, and the patient expired.

The Doctor believed that the spasm of the glottis occasioned by a forced depression of the tongue would not, of itself, have been sufficient to cause death in a strong and healthy person, but, in the present instance, the patient being under some emotion, an old cardiac had probably been awakened and a rapidly fatal congestion, or apoplexy of the lungs had followed. This opinion was, to some extent, borne out by the fact that the patient showed marks of scarified cups under the left breast.

In commenting on the above case, the editor of the Paris Medical (No. 46) observes that it accords with what he has long ago published on asphyxia caused by enforced immobility of the tongue. If a patient's mouth be immoderately opened by means of a gag, the tongue can no longer move, deglutition of saliva becomes impossible, and the larynx is immobilized. Under these conditions, rapidly fatal asphyxia can be produced. An instance of it has been observed. Satisfactory evidence may be obtained from the following experiment: Let the tongue be immobilized by placing a finger upon it, the mouth being opened or closed; or, let the jaws be kept wide open by a bit of wood inserted between the teeth, and the result will be a respiratory anguish which, if prolonged, would lead to asphyxia.—*American Journal of Obstetrics.*

**A Novel Suit Against a Doctor.**—A paper on the "Laws of Malpractice," by Dr. G. F. Souwers, contained in the Philadelphia Medical and Surgical Reporter, relates a novel case which took place in Michigan. A physician who had been engaged to attend a lady in confinement, took with him an assistant who was supposed by the patient and family to be a physician or a student, and who rendered aid in the delivery. It was aftewards discovered, to the consternation of the woman and her friends, that the assistant was simply a friend of the doctor, and had nothing to do with the study or practice of medicine, but, as was believed, had no other object than the gratification of a morbid curiosity. So great was the indignation against the doctor that a law suit was instituted on the charge of making an indecent and unprofessional exposure of his patient. The charge was sustained by the court and the doctor convicted. In this decision we heartily concur. A physician who would take the advantage of his professional privileges in that manner ought to be fined, imprissoned, and struck from the roll of the profession.—*Pacific Med. Journal.*

**Tetanus Cured.**—Dr. Layton reports in the New Orleans Medical Journal, a case of tetanus cured with the following prescription:

℞ Sulphate of eserine . . . . . . . . . . . . . . . . . . . . . . . . . gr. ½,

Pure glycerine, . . . . . . . . . . . . . . . . . . . . . . . . . . . . . . f. ʒij,

Syrup of orange flowers, . . . . . . . . . . . . . . . . . . . . . . f. ʒxiv,

Water, . . . . . . . . . . . . . . . . . . . . . . . . . . . . . . . . . . . . f. ℥ij.

M. S. Teaspoonful (1-64 grain or one milligramme of eserine) every hour. I should say here that I was advised by Mr. Lascar, the obliging Chemist of Messrs. I. L. Lyons & Co., to use the solution in glycerine, eserine being so easily decomposed otherwise. Even the short exposure to the air of the salt, during the time required for preparing a dose, is sufficient to cause an increase in weight of the eserine so exposed. Mr. Lascar has remarked that glycerine prevents the decomposition of the solution.

From January 10th, in the evening, the doses of eserine were given at intervals of an hour and a half; later, the time was increased to two hours; the remedy was continued until January 17, when the child had taken, in all, 3 grains of eserine; the prescription was then discontinued, the only remaining trace of the attack being some rigidity of the jaws, which had entirely disappeared by January 30th.

Eserine or physostigmia is an alkaloid obtained from calabar bean, and is an agent of great power, and should be given with caution.—[REC. ED.

**Permanence of the Scarlatinous Virus.**—Dr. G. T. Jenkins, of Keokuk, reports a very interesting case. A child was taken with this disease and died. There had been no known source of contagion. Upon inquiry it was learned that the parents had lost a child from this disease two years before, and that the only clothing saved had been a cap, made of woolen cloth, which had been packed away in a tin box, and that it was taken out and worn by the second child three days before he was taken sick. How long will the poison last?—*Med. and Surg. Reporter*

**Some of the Consequences of Phimosis and Adherent Prepuce.**—After brief reference to the antiquity of the custom of circumcision among the Jews and Egyptians, the writer (Editor Louisville Medical News) gives Dr. Sayre due credit for demonstrating these conditions as bearing a conclusive relation to irritability of the bladder and arrest of development of the lower extremities, etc., by reflex action, and proceeds to state that Dr. Sayre has not covered the whole ground.

"Dr. Barwell, in his Treatise on the Diseases of Joints (page 289) states that he has had forced upon his observation the coincidence of phimosis and hip-joint disease, which in his experience has been so frequent as to draw from him the opinion that it is not fortuitous, but is a physiological and potent relation—probably a cause to be ranked along with the strumous diathesis and local injury." His conclusions are based on a large number of cases and upon the observations of Mr. Baker, at Evelina Hospital, that Jews rarely have hip-joint disease.

"In Warren's Treatise on Hernia, just issued, there is quoted (page 17) an essay by Samuel Osborn, F. R. C. S., upon Phimosis as a Cause of Hernia in Infants." This essay was prompted by noticing the frequent co-existence of these conditions. "He thinks that the contracted preputial orifice offers such an impediment to the flow of urine that extraordinary efforts of straining are occasioned," etc.

"Again, Mr. Kempe is reported to have found that out of 50 cases of congenital phimosis, in 31 there was rupture."

"In the Alienist and Neurologist, for October, 1881, Dr. E. W. Saunders reports four cases of reflex gastralgia dependent upon adherent prepuce." The first of these was relieved, after failure with the usual remedies, by circumcision; the second, by detaching the prepuce. 'The other cases were of the same nature, though the family history was not so good."—*Louisville Medical News, January, 1882.*

**Paracentesis of the Bladder Through the Perineum and Prostate.**—Mr. Reginald Harrison (British Medical Journal and the Medical Record), after justly criticising the various methods of relieving a distended bladder in old men with enlarged prostate, describes a means of relief practiced by himself upon a man eighty-four years of age. He passed a trocar with its canula through the perineum and gland into the bladder so as to reach the point where this viscus is uncovered by peritoneum, guiding the instrument by the left forefinger in the rectum. He then removed the trocar and secured the canula, *in situ* by means of tapes.

The advantages he claims are: More complete drainage, and, therefore, less danger from pyelitis, cystitis, etc., than where a catheter is retained in the urethra; a short "low level" urethra more adapted to the new relation of prostate and bladder; a continuous discharge of urine at night, with easy command over the same during the day, the flow being regulated by a spring clamp fitted to a bit of rubber-tubing, which latter is connected with the canula.—*Medical Times.*

**Bad Effects of Quinine.**—Dr. Marion Sims, in some comments on a paper published in the New York Medical Gazette of October 22d, refers to the fact that as early as 1847 Dr. W. O. Baldwin, of Montgomery, Alabama, had detailed several cases in which severe symptoms followed the administration of quinine. One of particular interest is that of a lady who received eighty grains in a few hours for an attack of pernicious intermittent fever; of this she was relieved, but the next day was blind, and has remained so to the present moment, a period of thirty-five years.

In the accompanying article of Dr. Baldwin, several cases of death attributed by him to the use of large doses of the drug are related, and experiments on dogs illustrating its toxic properties cited. Dr. Baldwin found, chiefly, a remarkable increase in frequency of the pulse, dyspnœa, maximal pupillary dilatation, convulsions, and, in one case, furious delirium, the dog biting and barking at everything about him. His observation that the effect of quinine is proportionately far greater on puppies than on grown dogs, is in harmony with the known fact that relatively small doses may prove fatal to children.—*Ame. Jour. Neurol. Psychiatry, Feb. 1882.*

**Almost a Snake-Story.**—The Boston Medical and Surgical Journal says:

A moving story comes to us of the sufferings of an unfortunate lady in a village in the northern part of New York State, who was recently taken violently ill, and when the local physician arrived confided to him the startling intelligence that there was a snake in her stomach, which she had swallowed last September while drinking from a brook. The report goes on to state that the doctor, upon investigation, "became satisfied that the woman had swallowed a tadpole, which has since turned into a frog," and that the presence and movements of the reptile in the stomach can readily be felt from the outside. The patient is to be taken to the hospital in Albany for treatment, and the subsequent developments of the case will no doubt be awaited with no little interest. —*Medical News.*

**The Physician in Court**—Speaking of a recent trial in England, in which it was held that the surgeon in a certain case was forced to disclose the confidence of his patient, the Louisville Medical News (March 18) says:

"Those laws which compel a medical man to appear, and, upon pain of imprisonment, force him to divulge in open court the secrets of which he necessarily became possessed through his professional relations to his patient, are a relic of barbarity, a disgrace to the code, and should be erased from the statute books of all enlightened nations." Certainly the relations between patient and physician are as intimate as those existing between client and attorney. The laws of the country do not force a lawyer to disclose the statements of his client. The physician should not be forced to divulge the secrets of his patient.—*Va. Med. Monthly.*

